# Hepatic Vein Pylephlebitis as a Cause of Bilateral Pyopneumothorax

**DOI:** 10.7759/cureus.41039

**Published:** 2023-06-27

**Authors:** Beth Schwartz, Inderpal Singh, Harish Gidda, Leonard B Johnson

**Affiliations:** 1 Internal Medicine, Ascension St. John Hospital, Detroit, USA; 2 Infectious Diseases, Ascension St. John Hospital, Detroit, USA

**Keywords:** hepatic abscess, septic emboli, streptococcus milleri, pneumothorax (ptx), hepatic pylephlebitis

## Abstract

Pylephlebitis is a rare complication of intra-abdominal infections and has a significant mortality rate, necessitating early recognition for optimal treatment. Here, we present the case of a 36-year-old male with fever, shortness of breath, cough, and epigastric pain. He was ultimately diagnosed with hepatic vein pylephlebitis along with multiple pulmonary and hepatic lesions believed to be septic emboli and hepatic abscess. He developed recurrent bilateral pyopneumothorax which required drainage by interventional radiology multiple times. The patient improved and was discharged on intravenous antibiotics for four weeks. While hepatic abscesses are a known complication of pylephlebitis, pyopneumothorax is a rare, unreported complication. Recognition of this potential complication is important for clinicians when treating patients with hepatic vein pylephlebitis.

## Introduction

Pylephlebitis is an infective thrombosis of the hepatic vein which can complicate intra-abdominal or pelvic infections. Complications including instances of small bowel infarction and septic pulmonary emboli have been previously described [1]. However, pyopneumothorax has not been reported as a complication of hepatic vein pylephlebitis. Pylephlebitis has been reported in some studies to have a mortality rate ranging from 11% to 32%, making increased recognition of this complication important for clinicians [2,3].

## Case presentation

A 36-year-old male presented initially with symptoms of fever, dyspnea, productive cough with dark brown sputum, and epigastric pain. The patient had a past medical history significant for diverticulitis complicated by diverticular perforation, intra-abdominal abscess, and colo-vesicular fistula formation necessitating loop colostomy several years before admission. On examination, the patient exhibited tachypnea and tachycardia and appeared diaphoretic. Auscultation of the lungs showed diminished breath sounds on the right side, and no abdominal tenderness was noted on palpation. Initial laboratory evaluation was significant for elevated white blood cell count, total and direct bilirubin, alkaline phosphatase, and D-dimer (Table 1).

**Table 1:** Initial laboratory workup on admission. HIV = human immunodeficiency virus; HIV-1 = human immunodeficiency virus type 1; HIV-2 = human immunodeficiency virus type 2

Test	Results	Reference range
White blood cell count	47.48	4.00–11.00 × 10^3^/µL
Hemoglobin	10.9	12.0–16.0 g/dL
Platelet count	93	150–400 K/µL
D-dimer	2,640	<500 ng/mL
Creatinine	1.46	0.5–1.1 mg/dL
Sodium	132	135–145 mmol/L
Total bilirubin	3.7	0.1–1.2 mg/dL
Direct bilirubin	3.0	0.0–0.8 mg/dL
Alkaline phosphatase	281	20–120 IU/L
Albumin	2.3	3.5–5.0 g/dL
HIV antigen/antibody screen	Nonreactive	Nonreactive
HIV-1 p24 antigen	Nonreactive	Nonreactive
HIV-1 antibody	Nonreactive	Nonreactive
HIV-2 antibody	Nonreactive	Nonreactive

**Figure 1:**
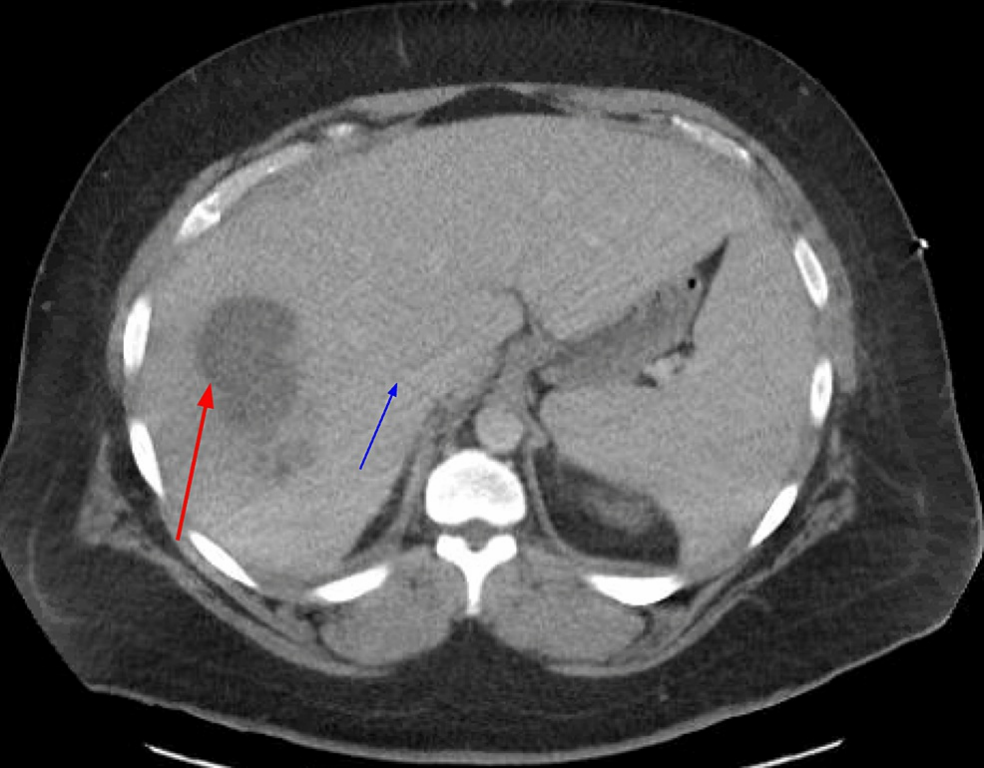
Abdominal computed tomography scan with intravenous contrast image showing hepatic abscess (red arrow) with a linear area of attenuation indicating opacification of a hepatic vein (blue arrow).

**Figure 2:**
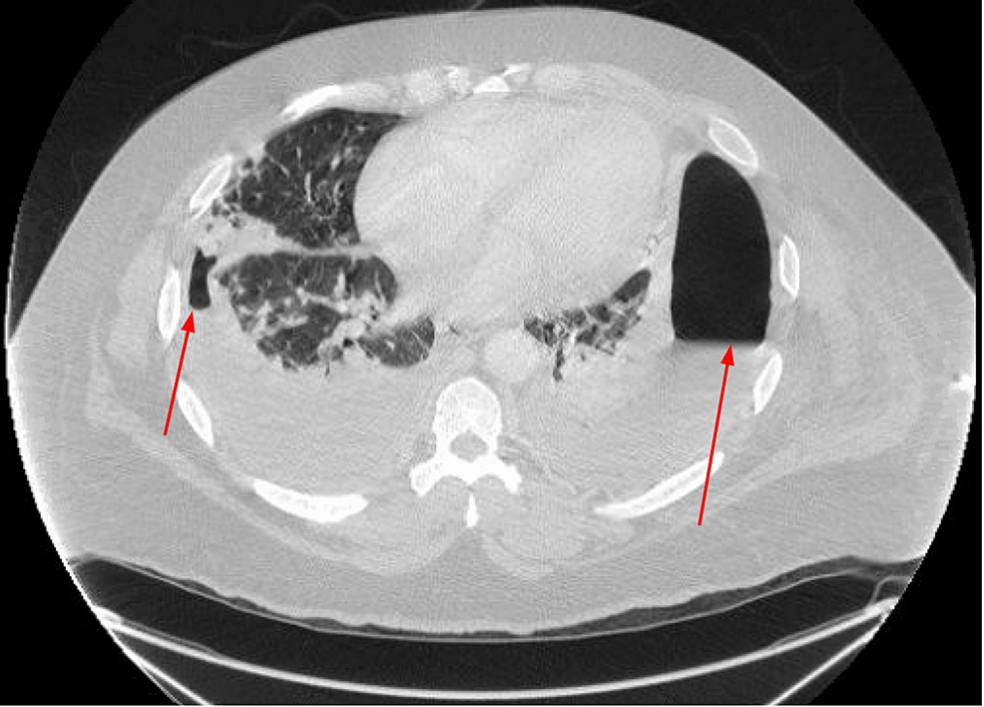
Thoracic computed tomography image demonstrating bilateral loculated pleural effusions with air in the left pleural space (red arrows).

A computed tomography (CT) scan with intravenous contrast of the abdomen, pelvis, and thorax demonstrated opacification of the hepatic vein indicative of thrombosis. A hepatic fluid collection measuring 9 cm transversely along with multiple cavitary lesions was also seen in the lung fields bilaterally, which were suggestive of hepatic abscess and septic pulmonary emboli, respectively (Figures 1, 2). A transthoracic echocardiogram was performed and was negative for vegetation. Empiric antibiotics were initiated with vancomycin, cefepime, and metronidazole. Blood cultures were obtained and grew pan-susceptible *Streptococcus anginosus.* As a result, antibiotic coverage was changed to ceftriaxone and metronidazole. Interventional radiology was consulted, and the hepatic abscess was subsequently drained. Abscess cultures grew *Escherichia coli* along with *Streptococcus constellatus* and *Fusobacterium necrophum*. A repeat CT scan of the thorax, abdomen, and pelvis showed a decrease in the size of the hepatic abscess, along with new, large bilateral pleural effusions. Bilateral thoracenteses were performed, and the fluid sample was sent for laboratory analysis and culture. Laboratory analyses were indicative of exudative pleural fluid with fluid cultures also positive for *S. anginosus*. The patient had serial thoracenteses to drain fluid collections and placement of a CT-guided drain. The patient’s clinical status improved over the course of the admission, and he was discharged on intravenous ceftriaxone and metronidazole for four weeks.

## Discussion

Hepatic vein pylephlebitis is a rare complication of intra-abdominal or pelvic infections in organs relying on the portal system for drainage [1]. Patients may present with abdominal pain and fever; however, the reported symptoms can vary and are often nonspecific. Diagnosis of pylephlebitis requires a thrombosis of the affected vein, and the patients are frequently bacteremic and febrile [1]. CT imaging is diagnostic if the overall case is consistent with a diagnosis of pylephlebitis [4]. Additionally, patients may have leukocytosis, although this is not required for diagnosis [1]. Abnormal alkaline phosphatase and gamma-glutamyl transferase are also common features [1]. Potential complications can include liver abscess, septic pulmonary emboli, bowel ischemia, and portal hypertension as long-term considerations [1]. Pyopneumothorax, as seen in this patient, has not been reported in the literature and is a rare complication of pylephlebitis. Additionally, the bacteria isolated in the blood and pleural fluid specimens obtained, *S. anginosus*, is part of a group of streptococci which was formerly thought to be commensal organisms that rarely cause infections in humans [5]. *E. coli *and *Bacteroides fragilis* appear to be common isolates identified in cases of pylephlebitis, although in a review conducted by Fusaro et al., *Streptococcus* species were responsible for up to 15% of cases [1]. Other cases of *Streptococcus* causing pyopneumothorax exist; however, they appear to be secondary to a primary lung infection unrelated to pylephlebitis [6].

Management of pylephlebitis should include antimicrobial coverage for gram-negative and anaerobic bacteria, usually accomplished with metronidazole and a third-generation cephalosporin or fluoroquinolone [1]. Antibiotics are recommended for four to six weeks, with parenteral treatment until significant clinical improvement is appreciated, commonly around two to three weeks into the patient’s disease process [1,3]. The utility of anticoagulation in patients suffering from pylephlebitis has been debated as no randomized controlled trials exist to examine this question. Anticoagulation has been thought to be beneficial for patients who have continued fever while on the correct antimicrobial regimen and those with the propagation of thrombosis, though data on the duration of therapy are also lacking [1]. A review of 91 cases conducted by Choudry et al. found a lower mortality rate in patients treated with anticoagulation compared to antimicrobial therapy alone [2]. Similar findings were observed by Kanellopoulou et al. wherein on reviewing 81 cases, anticoagulation was associated with a higher rate of complete vein recanalization and a lower mortality rate [7].

## Conclusions

Pyopneumothorax secondary to hepatic vein pylephlebitis has not been reported in the literature. The bacteria implicated in this case, *S. anginosus*, has been identified in other cases of pyopneumothorax, however, not in relation to pylephlebitis. Given the significant mortality rate of pylephlebitis, clinicians should be aware of this condition and its complications to improve patient outcomes.
